# Intermittent kangaroo mother care and the practice of breastfeeding late preterm infants: results from four hospitals in different provinces of China

**DOI:** 10.1186/s13006-020-00309-5

**Published:** 2020-07-17

**Authors:** Bo Zhang, Zhiying Duan, Yingxi Zhao, Sarah Williams, Stephen Wall, Limin Huang, Xiaoqin Zhang, Wenli Wu, Jieya Yue, Lin Zhang, Jun Liu, Gengli Zhao

**Affiliations:** 1 Peking University First Hospital, 1 Xi’anmen St, Xicheng, Beijing, China; 2 University of Oxford Nuffield Department of Medicine, Oxford, UK; 3 Save the Children UK, London, UK; 4 Save the Children Saving Newborn Lives, Washington, DC USA; 5 Hunan Provincial Maternal and Child Health Hospital, Changsha, Hunan China; 6 Northwest Women & Children Hospital Department of Obstetrics, Xi’an, Shaanxi China; 7 Linyi Maternity and Child Health Hospital, Linyi, Shandong China; 8 Save the Children, Chengdu, China

**Keywords:** Kangaroo mother care, Skin-to-skin contact, Breastfeeding, Late preterm infants

## Abstract

**Background:**

China has an extremely low exclusive breastfeeding rate. Kangaroo mother care (KMC) has been shown to increase the exclusive breastfeeding rate among infants born extremely or very preterm. However, there is limited evidence surrounding intermittent KMC and exclusive breastfeeding in late preterm infants. In our study we investigated the association between the provision of intermittent KMC and breastfeeding practice for late preterm infants in four hospitals in different provinces of China.

**Methods:**

Intermittent KMC was recommended to the mothers of all preterm infants admitted to the postnatal wards of participating hospitals between March 2018 and March 2019. Those who agreed to practice KMC were enrolled in the “KMC group”, those who did not were enrolled in the “No KMC group”. Basic maternal socio-demographic information was collected, feeding practice; outcome and method, were recorded daily whilst in hospital. A follow-up survey of feeding practice was conducted 42 days after discharge. Calculations for feeding practice were performed separately for both groups. Logistics regression was used to analyze the association between KMC and feeding outcome and method, adjusting for socio-demographic covariates.

**Results:**

Among the 844 mothers participating in the study, 627 (74.3%) chose to perform KMC. More of the mothers who provided KMC were exclusively breast milk feeding their infants in the 24 h before hospital discharge (54.6%) and at follow-up (57.3%), compared to mothers who did not provide KMC (34.6% at discharge and 33.2% at follow-up,). Mothers in the KMC group were more likely to be breastfeeding (method) than mothers in the No KMC group (65.3% vs. 52.1% at discharge, and 83.1% vs. 67.3% at follow up). Logistic regression indicated that compared with the No KMC group, mothers who provided KMC were twice as likely to be exclusively breast milk feeding their infants at discharge (OR = 2.15 (95% CI 1.53, 3.02)), use breastfeeding method at discharge as opposed to other means such as bottle or cup feeding (OR = 1.61 (95% CI 1.15, 2.25)), be exclusive breast milk feeding at follow-up (OR = 2.55 (95% CI 1.81, 3.61)), and use breastfeeding method at follow-up (OR = 2.09 (95% CI 1.44, 3.02)).

**Conclusions:**

Intermittent KMC was associated with a nearly doubled increase in exclusive breast milk feeding (outcome) and breastfeeding (method) at both discharge and 42 days after discharge for late preterm infants. This is especially important in China where exclusive breastfeeding rates are low, intermittent KMC provides a feasible means to increase the likelihood of these vulnerable infants receiving the benefits of exclusive breastmilk.

## Background

Kangaroo mother care (KMC) is a cost-effective intervention recommended by the World Health Organization (WHO) for the care of stable preterm infants [1]. Infants are cared for skin-to-skin on the chest of their mother or another caregiver and receive exclusive breastfeeding (ideally) or breast milk feeding. Compared to conventional care it has been shown to reduce mortality, the incidence of severe infection and hypothermia and improve health outcomes including exclusive breastfeeding. KMC has been found to increase the likelihood of exclusive breastfeeding at hospital discharge by 50% and at 1 to 4 month follow-up by 39% [2].

Where continuous KMC is not possible WHO recommends intermittent KMC over conventional care for stable newborns weighing 2000 g or less at birth [1]. Intermittent KMC refers to recurrent but not continuous skin to skin contact between a mother and infant alternated with conventional care. Evidence around intermittent KMC and mortality is inconclusive however there is evidence that intermittent KMC reduces the risk of hypothermia, severe infection and nosocomial infection [3]. A randomized control trial in India suggested that early intermittent KMC increased exclusive breast milk feeding and direct breastfeeding in low birth weight infants [4].

The breastfeeding rate in China is low, for both term and preterm infants. According to a survey of over 10,000 mothers in 2018, the rate of exclusive breastfeeding for infants under 6 months of age was 29.2%, and only 11.3% of mothers breastfed their infants within an hour of birth [5]. While the survey did not disaggregate breastfeeding rate based on gestational age at birth or birth weight, a more recent observational study with a smaller sample size indicated an exclusive breastfeeding rate of 22.5% at 6 months in infants born preterm [6]; lower than their full-term counterparts. Barriers to exclusive breastfeeding of preterm infants in China include low levels of maternal breastfeeding self-efficacy (a mother’s belief in her ability to breastfeed) and symptoms of postpartum depression amongst mothers [6]. Health care providers were found to have limited knowledge of breastfeeding preterm infants and the specific benefits of breast milk for these infants, this hindered their ability to support new mothers [7]. There is empirical evidence of the benefit of breast milk for preterm infants including improved cardiovascular function in adulthood [8].

There is published evidence to demonstrate that KMC increases the exclusive breastfeeding rate amongst preterm infants, however the majority of these studies are set in neonatal units with extremely preterm or very preterm infants. The effect of intermittent KMC on the breastfeeding outcome of late preterm infants cared for on busy postnatal wards where the length of hospital stay is relatively short and therefore the amount of health worker support for KMC is also limited has not previously been investigated. In addition, most of the evidence around KMC and exclusive breastfeeding has been generated from outside the Western Pacific region [2, 9, 10] and there have been no studies found conducted in China.

This study is part of a larger piece of implementation research on the feasibility of introducing intermittent KMC into China’s postnatal and neonatal wards, understanding the acceptance of KMC by nurses and subsequent uptake by mothers. In the current analysis we aim to determine the effect of intermittent KMC exposure for late preterm infants in a postnatal ward setting on exclusive breast milk feeding (outcome) and breastfeeding (method) at hospital discharge and at 42 days post discharge follow-up.

## Methods

### Study design and population

In 2017, as part of the larger KMC implementation research project, standardized guidelines for KMC were developed along with procedural information, training material and data capture tools these were then piloted and finalized. Training on KMC, the study protocol and tools were provided to medical and nursing staff working on the neonatal units and postnatal wards. Data collection took place from March 2018 to March 2019. During this time two meetings were held with staff from the participating wards in order to ensure that data was consistently and reliably collected in all sites.

This current analysis is based on the data collected from the four participating postnatal wards all located in level-III hospitals in different provinces of Southeast and Northwest China. The approximate number of births per month in each hospital ranged from 500 to 1000 including both full-term and preterm infants. The standard procedure is for all preterm infants to be assessed by a pediatrician at birth, late preterm infants with stable vital signs are then admitted to postnatal wards with their mothers. Late preterm infants were defined as those born between 34 weeks and 36 weeks and 6 days of gestation. Gestational age was determined by use of a dating scan in pregnancy. The postnatal wards practice rooming in, and non-separation of mothers and infants. The majority of rooms on postnatal wards have space for two beds, however some are single bedded, and some have multiple beds, curtains are provided for privacy. The length of hospitalization is determined by the mode of delivery and the condition of the patients, ranging from one to 4 days. Most women and infants who experience vaginal birth are discharged after 1 day.

**Fig. 1:**
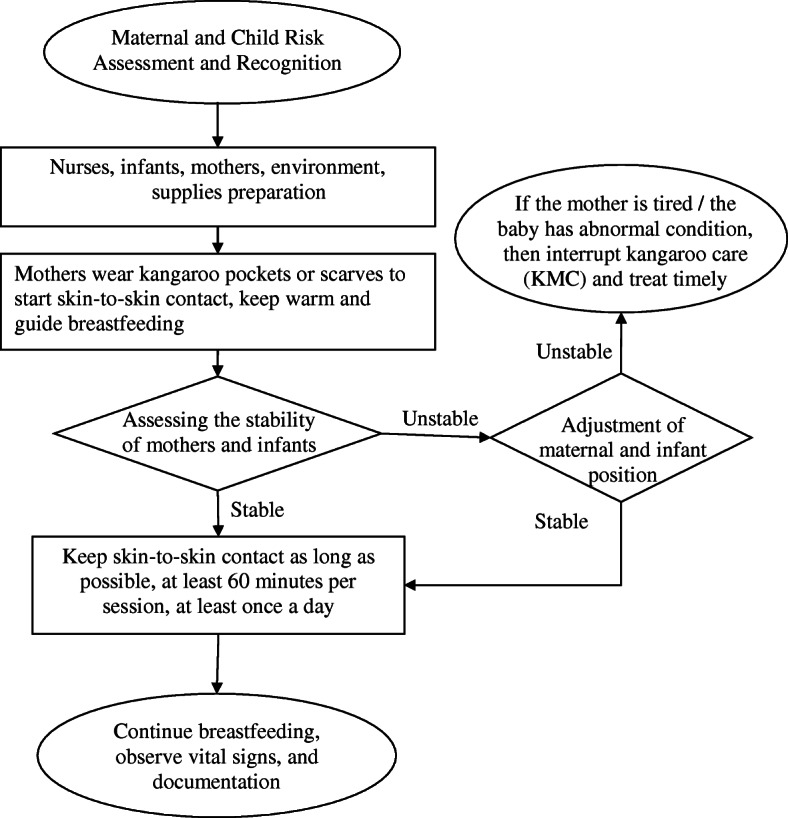
Operational process for Kangaroo mother care on postnatal wards

KMC was recommended to the mothers and families of preterm infants on the postnatal wards. Exclusive breastfeeding was also recommended for all infants regardless as to whether mothers chose to provide KMC. The study was explained to mothers and families. In total 1007 mother and infant pairs were enrolled in the study. Preterm twins and their mother were recorded as two separate pairs in the analysis. Those who chose to provide KMC were termed the “KMC group” (*N* = 752) and those who chose not to provide KMC were called the “No KMC group” (*n* = 255). The specific KMC provision flow diagram is shown in Fig. 1.

Basic maternal socio-demographic information was collected using a structured survey along with obstetric history including maternal parity, presence of obstetric complications and mode of delivery. The infants’ birthweight and gestational age were recorded. During hospital stay feeding outcome and method were recorded daily, and each episode of KMC provision was recorded and its length noted. At hospital discharge feeding practice (outcome and method) for the preceding 24 h was recorded. A follow-up survey was conducted by phone for all participants 42 days after hospital discharge. The survey included questions about feeding practice (outcome and method) provision of KMC after discharge, the incidence of serious disease in the mother or infant and the current weight of the infant.

Ethical approval for the study was obtained from Peking University First Hospital Biomedical Research Ethics Committee. All participants gave written consent.

### Measures and variables

The main outcome variables analyzed were the feeding outcome and the feeding method of preterm infants 24 h before discharge (documented by the nurses) and 42 days after discharge (self-reported by the mothers). Feeding outcome refers to the make-up of the infant’s feeds, classified as “exclusive breastmilk feeding” (in accordance with WHO’s definition [11]), “exclusive formula feeding” or “mixed feeding” (when a baby receives both breast and formula milk), while feeding method refers to the way in which infants receive their milk, this was classified as either “breastfeeding” (breastfeeding, or breastfeeding and other methods of feeding) or “other” which included infants fed only via bottle, tube, syringe or cup and no breastfeeding.

The independent variable in the analysis was KMC vs. No KMC. We also included other socio-demographic indicators as exposure variables in the study, including maternal age, education attainment (high school, college, university and above), occupation (including government employee, technician and worker), parity (primipara or multipara), pregnancy-related complications (yes, or no), infant’s birth weight (> = 2500 g, or < 2500 g) and gestational age (36 weeks - 36 weeks and 6 days, or 34 weeks - 35 weeks and 6 days). Additionally, for infants receiving KMC, we documented the number and duration of KMC sessions during hospital stay.

### Statistical analysis

Selected socio-demographic and delivery-related variables were considered separately for the KMC and No KMC groups. The average KMC frequency and KMC duration were ascertained for the KMC group only. To compare the difference in feeding outcome and feeding method between KMC and No KMC groups, the unadjusted percentage of breastfeeding outcome and method at discharge and 42 days after discharge was calculated.

Logistics regression was used to analyze the association between KMC and feeding outcome and method. All models were commonly adjusted for covariates including age, education, occupation, parity, pregnancy-related complications, mode of delivery, birthweight and gestational age. Odds ratios for feeding outcome at discharge and at follow-up, and feeding method, were reported within KMC and No KMC groups. All statistical analyses were performed using Stata V14, and SAS V.9.3, and test results were reported to be significant at 0.05 level.

## Results

Table 1 presents the general characteristics of the study population separated by KMC group and No KMC group. The analysis excludes those who could not be successfully followed-up (*N* = 153), 116 were from the KMC group and 37 from the No KMC group. The follow-up rates were similar in both groups (KMC group 84.6% vs. No KMC group 85.5%). A total of 844 participants were included in the final analysis, of these 627 (74.3%) chose to provide KMC on the postnatal ward. Mothers in the KMC and No KMC groups were found to be similar in terms of age, educational attainment and parity. Compared with the KMC group (56.3%), there were more mothers with pregnancy-related complications in the No KMC group (72.8%). Similarly, more mothers delivered through cesarean section in the No KMC group (69.6%) than in the KMC group (60.5%). In the KMC group 12.8% of mothers gave birth before completing 36 weeks of pregnancy, compared with 3.2% of mothers in the No KMC group. KMC was provided an average of 3.5 times before hospital discharge and the average duration each time was 65.8 min.

**Table 1:** Basic characteristics of the study population by kangaroo mother care group

	Kangaroo mother care (*N* = 627)	No Kangaroo mother care (*N* = 217)	*p* value
Age			0.213
< 30	212 (33.81%)	87 (40.09%)	
30–34	234 (37.32%)	77 (35.48%)	
> =35	181 (28.87%)	53 (24.42%)	
Educational attainment			0.514
High school	205 (32.70%)	67 (30.88%)	
College	152 (24.24%)	47 (21.66%)	
University & above	270 (43.06%)	103 (47.47%)	
Parity			0.804
Primipara	387 (61.72%)	136 (62.67%)	
Multipara	240 (38.28%)	81 (37.33%)	
Pregnancy-related complications			< 0.001*
No	274 (43.70%)	59 (27.19%)	
Yes	353 (56.30%)	158 (72.81%)	
Delivery mode			0.016*
Vaginal delivery	248 (39.55%)	66 (30.41%)	
C-section	379 (60.45%)	151 (69.59%)	
Birth weight			0.133
Normal (> = 2500)	545 (86.92%)	197 (90.78%)	
Low weight (< 2500)	82 (13.08%)	20 (9.22%)	
Gestational age			< 0.001*
36–36 + 6 weeks	547 (87.24%)	210 (96.77%)	
34–35 + 6 weeks	80 (12.76%)	7 (3.23%)	
Average KMC frequency before discharge (mean ± SD)	3.47 (1.74)	–	–
Average KMC duration before discharge (minutes, mean ± SD)	65.75 (58.27)	–	–

Note: Values are n (%) unless otherwise specified; *refers to significant at 0.05 level

**Fig. 2:**
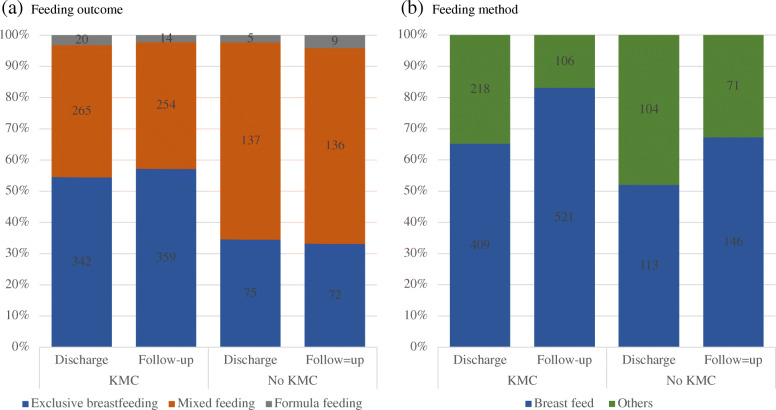
Feeding outcome (**a**) and methods (**b**) at discharge and at follow-up

Figure 2 compares feeding outcome and method at discharge and at follow up between the two groups. More mothers in the KMC group practiced exclusive breast milk feeding at discharge (54.6%) and at follow-up (57.3%), compared with mothers in the No KMC group (34.6% at discharge and 33.2% at follow-up,). The majority of mothers in the No KMC group were mixed feeding at discharge (63.1%) and at follow up (62.7%). In both groups very few mothers provided formula milk only. Both groups experienced an increase in breastfeeding rate at follow up. Mothers in the KMC group were more likely to be breastfeeding at discharge compared to mothers in the No KMC group (65.3% vs. 52.1% respectively) and at follow-up (83.1% vs. 67.3%). The increase in breastfeeding between discharge and follow up was larger in the KMC group than in the No KMC group.

Table 2 illustrates the results of the logistics regression, analyzing the association between selected variables, feeding outcome, and feeding method at discharge and follow-up. After adjusting for other co-variates including age, education, occupation, parity, presence of complications, mode of delivery, birth weight and gestational age, intermittent KMC was significantly associated with an increased likelihood of exclusive breast milk feeding (outcome) and breastfeeding (method). Compared with mothers in the No KMC group, mothers in the KMC group were twice as likely to provide exclusive breast milk feeding at discharge (OR = 2.15 (95% CI 1.53, 3.02)), and breastfeeding (method) at discharge (OR = 1.61 (95% CI 1.15, 2.25)), provide exclusive breast milk feeding at follow up (OR = 2.55 (95% CI 1.81, 3.61)), and breastfeeding (method) at follow up (OR = 2.09 (95% CI 1.44, 3.02)). It was observed that educational attainment and parity did not significantly influence breastfeeding practice, older mothers (over 30 years of age) were less likely to exclusively breastfeed at follow-up than their younger counterparts. Mothers with complications were less likely to perform breast feeding (method) (OR = 0.69 (95% CI 0.51, 0.94) at discharge, OR = 0.68 (95% CI 0.47, 0.99) at follow-up), though our results indicate that it does not influence breast milk feeding (outcome).

**Table 2:** Odds ratio of the association between selected variables and exclusive breastmilk feeding (outcome), breast feed (method) at discharge and at follow-up

	Exclusive breastmilk feeding (outcome) at discharge	Breast feed (method) at discharge	Exclusive breastmilk feeding (outcome) at follow-up	Breast feed (method) at follow-up
Group				
No KMC	1.00	1.00	1.00	1.00
KMC	2.15 (1.53, 3.02) *	1.61 (1.15, 2.25) *	2.55 (1.81, 3.61) *	2.09 (1.44, 3.02) *
Age				
< 30	1.00	1.00	1.00	1.00
30–34	0.84 (0.60, 1.19)	0.98 (0.69, 1.40)	0.70 (0.49, 1.00) *	0.69 (0.46, 1.05)
> =35	0.99 (0.67, 1.46)	1.06 (0.71, 1.58)	0.64 (0.43, 0.95) *	0.82 (0.51, 1.32)
Educational attainment				
High school	1.00	1.00	1.00	1.00
College	1.19 (0.79, 1.80)	1.29 (0.85, 1.95)	0.77 (0.51, 1.17)	0.69 (0.43, 1.12)
University & above	1.30 (0.86, 1.98)	1.80 (1.17, 2.76) *	0.72 (0.47, 1.10)	1.04 (0.63, 1.71)
Parity				
Primipara	1.00	1.00	1.00	1.00
Multipara	1.21 (0.88, 1.65)	0.88 (0.64, 1.21)	1.29 (0.94, 1.78)	1.02 (0.70, 1.49)
Pregnancy-related complications				
No	1.00	1.00	1.00	1.00
Yes	1.00 (0.75, 1.35)	0.69 (0.51, 0.94) *	0.81 (0.60, 1.10)	0.68 (0.47, 0.99) *
Delivery mode				
Vaginal delivery	1.00	1.00	1.00	1.00
C-section	0.81 (0.60, 1.10)	1.09 (0.80, 1.49)	0.51 (0.38, 0.70) *	0.84 (0.58, 1.22)
Birth weight				
Normal (> = 2500)	1.00	1.00	1.00	1.00
Low weight (< 2500)	0.55 (0.35, 0.86) *	0.46 (0.29, 0.72) *	0.44 (0.27, 0.70) *	1.41 (0.78, 2.53)
Gestational age				
36–36 + 6 weeks	1.00	1.00	1.00	1.00
34–35 + 6 weeks	1.57 (0.97, 2.56)	1.50 (0.89, 2.54)	1.61 (0.97, 2.27)	2.16 (1.00, 4.64) *

Note: Additionally, adjusted for occupation; *refers to significant at 0.05 level

## Discussion

This study is the largest and one of only a few studies on KMC and breastfeeding in China. Our analysis shows that KMC was associated with a nearly two-fold increase in exclusive breast milk feeding (outcome) and breastfeeding (method) at both discharge and follow up in late preterm infants. Our results suggest that for late preterm infants, cared for on postnatal wards with their mothers, relatively brief exposure to intermittent KMC in hospital was associated with increased exclusive breastfeeding at discharge and at follow up.

Breastfeeding is routinely promoted to mothers during pregnancy and during their hospital stay in all participating hospitals in this study. This includes breastfeeding knowledge; the definition of exclusive breastfeeding, benefits of exclusive breastfeeding for 6 months, and positions for breastfeeding. The definition of exclusive breastfeeding is in accordance with the WHO’s definition where no other food or drink, not even water, except breast milk is allowed [11]. Nurses will observe mothers breastfeeding their baby daily during hospital stay and provide advice as needed. Despite this ongoing effort, the exclusive breastfeeding rate in China remains low (29.2% at 6 months) [5]. Our results suggest that intermittent KMC may help combat the low breastfeeding rates for preterm infants.

There is a greater likelihood that preterm infants will commence breastfeeding later and cease breastfeeding earlier than infants born at term [12], due to a series of barriers including but not limited to a lack of or perceived lack of adequate breast milk, an infant’s immature uncoordinated sucking and swallowing mechanism and an increased likelihood of maternal symptoms of depression due to preterm delivery [6, 13, 14]. This also applies to late preterm infants [15, 16]. Inadequate milk intake contributes to slow weight gain and protracted jaundice in late preterm infants, making routine formula supplementation and early termination of breastfeeding more likely [17].

Our study results are consistent with previous evidence from published literature that frequent skin-to-skin contact between mother and infant is crucial to the successful transition to direct breastfeeding in preterm infants [9, 14] and initiation of exclusive breastfeeding in healthy full-term infants [18]. Early skin-to-skin contact, within the first hour of birth, if possible, facilitates maternal milk production [19, 20]. While continued skin-to-skin contact on a daily basis accelerates neurophysiological development of the preterm infant [21], which contributes to establishment of an effective suckling mechanism. KMC on postnatal wards minimizes maternal-infant separation time and promotes increased breastfeeding [6, 22].

It is noteworthy that KMC may play a role in alleviating stress related to preterm birth, encouraging mothers to care for their late preterm infants and breastfeed. Parents of late preterm infants are likely to exhibit a lack of confidence and some may exhibit distress during feeding or symptoms of depression [23–25]. A previous study on breastfeeding outcomes for preterm infants in China suggests that mothers of preterm infants perceived their ability to breastfeed to be low and were more likely to have symptoms of depression, contributing to the unsuccessful establishment of breastfeeding [6]. It is likely that KMC encourages mothers, helps relieve stress and boosts their confidence, this in addition to the breastfeeding support provided by nurses and health professionals could positively contribute to the successful initiation of breastfeeding in late preterm infants.

Our analyses indicate that intermittent KMC on postnatal wards, even for a relatively short duration, may have an impact on exclusive breastfeeding, not only at discharge, but up to 42 days after discharge. Given the extremely low exclusive breastfeeding rate (29.2%) at 6 months of age in China [5], our study has strong public health implications. KMC is recommended for infants under 2000 g, however use of intermittent KMC for late preterm infants on postnatal wards could be encouraged by health professionals in order to improve breastfeeding outcomes.

There is limited research regarding KMC in late preterm infants breastfeeding especially infants who are not low birth weight. According to our literature search, previous studies either did not report the characteristics of KMC (continuous vs. intermittent) [26] or did not analyze breastfeeding patterns [27]. We have identified two studies with similarities to ours. Nyqvist (2008) reported the association between KMC duration with breastfeeding rate in 128 healthy late preterm (average weight 2.9 kg)-parent infant pairs in Sweden, however a significant association was only found for fathers providing KMC and not mothers [20]. A randomized controlled trial conducted by Hake-Brooks and Anderson in the US in 2008 included 66 preterm infants born at different gestations (47% were of 36 weeks gestational age) and reported a nearly doubled increase in the exclusive breastfeeding rate (72% vs. 60% at discharge, 33% vs. 17% at 6 weeks, 19% vs 3% at 3 months, 8% vs. 0% at 6 months) though they did not disaggregate their analysis by gestational age and half of the infants were cared for on neonatal intensive care units [28]. To reach a firm conclusion regarding the effect of intermittent KMC on breastfeeding in heavier late preterm infants, further studies are needed.

Our study adds value to the global literature on KMC. Firstly the study population, late-preterm infants, is a population understudied and often overlooked in KMC literature. Secondly our study suggests that a relatively brief period of intermittent KMC is associated with significant increases in exclusive breastfeeding rates. If our findings were corroborated by more rigorous research design for example quasi-experimental study in the future, a strong recommendation for intermittent KMC for late preterm infants in similar settings could be made as a low cost and feasible solution to increasing breastfeeding rates. Lastly, considering the extremely low exclusive breastfeeding rate in China, KMC promotion for late preterm infants on postnatal wards may be an effective and feasible strategy to increase breastfeeding rates in China, which could have huge public health impact including but not limited to improved newborn health outcomes.

We recognize that our study has several limitations. Firstly it is not a randomized controlled trial., as KMC is known to be beneficial for preterm infants it was deemed unethical to randomize mothers and infants to a group where they would not be encouraged to practice KMC. It is also possible that those who opted to provide KMC may have been more likely to breastfeed their infants than those who chose not to provide KMC; however, we found no significant difference in socio-demographic characteristics (including age and educational attainment) between the two groups. Moreover, the exclusive breastfeeding rate for mothers who did not provide KMC (33.2% at 42 days follow-up) is similar to the national exclusive breastfeeding rate at 6 months of age (29.2%); therefore, we believe our finding that KMC was associated with an increase in breastfeeding rates is valid. Nonetheless, this is a cross-sectional study and part of a larger piece of implementation research on the introduction of KMC to China’s postnatal and neonatal wards, as such the primary aim of the overarching study was not to access the effectiveness of KMC to improve breastfeeding rates but to investigate the feasibility of intermittent KMC on postnatal and neonatal wards and understand the resulting use of KMC by mothers, thus the interpretation of the results should be made with caution without implying any causal inference.

Secondly we noted a difference in the KMC vs. No KMC ratio across the four participating hospitals. In one hospital almost all of the participating mothers chose to provide KMC to their infants. We performed an additional analysis excluding this hospital in order to ensure it had not skewed the results, results of this analysis can be found in the Additional files 1 and 2. When we excluded this hospital, compared with mothers in the No KMC group, mothers in the KMC group were still nearly twice as likely to perform exclusive breast milk feeding at discharge (OR = 1.61 (95% CI 1.13, 2.31)), and breastfeeding (method) at discharge (OR = 1.31 (95% CI 0.92, 1.85)), be providing exclusive breast milk feeding at follow-up (OR = 2.51 (95% CI 1.74, 3.62)), and breastfeeding (method) at follow-up (OR = 2.61 (95% CI 1.74, 3.93)). As the four hospitals enrolled in the study are all tertiary hospitals with minimal differences in service delivery capacity, we felt it appropriate and useful to compare breastfeeding patterns between mothers and infants in different hospitals, thus we included all four hospitals in our analysis. Given the opportunity we would like to conduct further research to identify any differences between the hospital with the highest uptake of KMC and the other three in order to assess if changes could be made that would increase KMC uptake for preterm newborns.

Thirdly mothers and infants who could not be successfully followed-up were excluded from the analysis, this may have led to selection bias. However, the loss to follow up rate was similar between the KMC and the No KMC group (15.4% vs. 14.5%). Within the KMC group, those lost to follow-up reported a higher exclusive breastfeeding rate at discharge than those who were successfully followed-up (60.0% vs. 54.6%), while within the No KMC group those who were lost to follow up reported a lower rate than those who were successfully followed-up (23.5% vs. 34.6%). This suggests that the results of our analysis may underestimate the association between intermittent KMC and improved breastfeeding outcomes, the actual impact may be greater.

Lastly, the major outcome variables of breastfeeding were only verifiably collected at hospital discharge and self-reported at 42 days follow up. The study would have benefited from a longer term outcome variable, e.g. exclusive breastfeeding at 6-months of age, in order to provide a more robust clinical and public health recommendation.

## Conclusion

In this observational study, we found that KMC was associated with a nearly two-fold increase in exclusive breast milk feeding (outcome) and breastfeeding (method) at both discharge and at 42 days follow up in late preterm infants. We believe this suggests the potential benefit of even “low-dose” intermittent kangaroo mother care for late preterm infants. Additionally, considering China’s extremely low exclusive breastfeeding rates, KMC promotion for late preterm infants on postnatal wards may be an effective and a feasible strategy to increase breastfeeding rates for these infants in China.

## Supplementary information

**Additional file 1 eTable 1.** Basic characteristics of the study population by hospital.

**Additional file 2 eTable 2.** Odds ratio of the association between selected variables and exclusive breastfeeding (ingredient), breast feed (method) at discharge and at follow-up in selected three hospitals (excluding hospital A).

## Data Availability

The datasets used and/or analyzed during the current study are available from the corresponding author on reasonable request.

## Abbreviation

KMC: Kangaroo mother care
