# Supplementary material for: Intermittent kangaroo mother care and the practice of breastfeeding late preterm infants: results from four hospitals in different provinces of China
Source: Int Breastfeed J. 2020 Jul 17;15:64. doi: 10.1186/s13006-020-00309-5 (PMC7367356; doi:10.1186/s13006-020-00309-5)
Supplement: Supplementary file 1 — Additional file 1 eTable 1. Basic characteristics of the study population by hospital. [file 13006_2020_309_MOESM1_ESM.docx]

# **The Effect of Intermittent Kangaroo Mother Care on Breastfeeding Practice of Late Preterm Infants in China**

# Additional file 1

**eTable 1. Basic characteristics of the study population by hospital**

|  | Hospital A  (N=181) | Hospital B  (N=302) | Hospital C  (N=195) | Hospital D  (N=166) |
| --- | --- | --- | --- | --- |
| Age |  |  |  |  |
| < 30 | 58 (32.04%) | 120 (39.74%) | 98 (50.26%) | 23 (12.86%) |
| 30-34 | 75 (41.44%) | 120 (39.74%) | 57 (29.23%) | 59 (35.54%) |
| >=35 | 48 (26.52%) | 63 (20.53%) | 40 (20.51%) | 84 (50.60%) |
| Education attainment |  |  |  |  |
| High school | 39 (21.55%) | 77 (25.50%) | 132 (67.69%) | 24 (14.46%) |
| College | 49 (27.07%) | 85 (28.15%) | 40 (20.51%) | 25 (15.06%) |
| University & above | 93 (51.38%) | 140 (46.36%) | 23 (11.79%) | 117 (70.48%) |
| Parity |  |  |  |  |
| Primipara | 133 (73.48%) | 200 (66.23%) | 84 (43.08%) | 106 (63.86%) |
| Multipara | 48 (26.52%) | 102 (33.77%) | 111 (56.92%) | 60 (36.14%) |
| Pregnancy-related complications |  |  |  |  |
| No | 94 (51.93%) | 83 (27.48%) | 107 (54.87%) | 49 (29.52%) |
| Yes | 87 (48.07%) | 219 (72.52%) | 88 (45.13%) | 117 (70.48%) |
| Delivery mode |  |  |  |  |
| Vaginal delivery | 56 (30.94%) | 103 (34.11%) | 94 (48.21%) | 61 (36.75%) |
| C-section | 125 (69.06%) | 199 (65.89%) | 101 (51.79%) | 105 (63.25%) |
| Birth weight |  |  |  |  |
| Normal (>=2500) | 174 (96.13%) | 286 (94.70%) | 167 (85.64%) | 115 (69.28%) |
| Low weight (<2500) | 7 (3.87%) | 16 (5.30%) | 28 (14.36%) | 51 (30.72%) |
| Gestational week |  |  |  |  |
| 36 weeks | 144 (79.56%) | 297 (98.34%) | 165 (84.62%) | 151 (90.96%) |
| Less than 36 | 37 (20.44%) | 5 (1.66%) | 30 (15.38%) | 15 (9.04%) |
| % of mother enrolled for KMC | 180 (99.45%) | 127 (42.05%) | 173 (88.72%) | 147 (88.55%) |
| Average KMC frequency before discharge (mean ± SD) | 3.68 (1.75) | 3.88 (1.67) | 2.46 (1.15) | 4.03 (1.89) |
| Average KMC duration before discharge (minutes, mean ± SD) | 69.52 (21.85) | 67.52 (29.09) | 63.34 (103.03) | 62.55 (23.05) |
