# Supplementary material for: Intermittent kangaroo mother care and the practice of breastfeeding late preterm infants: results from four hospitals in different provinces of China
Source: Int Breastfeed J. 2020 Jul 17;15:64. doi: 10.1186/s13006-020-00309-5 (PMC7367356; doi:10.1186/s13006-020-00309-5)
Supplement: Supplementary file 2 — Additional file 2 eTable 2. Odds ratio of the association between selected variables and exclusive breastfeeding (ingredient), breast feed (method) at discharge and at follow-up in selected three hospitals (excluding hospital A). [file 13006_2020_309_MOESM2_ESM.docx]

# **The Effect of Intermittent Kangaroo Mother Care on Breastfeeding Practice of Late Preterm Infants in China**

# Additional file 2

**eTable 2. Odds ratio of the association between selected variables and exclusive breastfeeding (ingredient), breast feed (method) at discharge and at follow-up in selected three hospitals (excluding hospital A)**

|  | Exclusive breastmilk feeding (outcome) at discharge | Breast feed (method) at discharge | Exclusive breastmilk feeding (outcome) at follow-up | Breast feed (method) at follow-up |
| --- | --- | --- | --- | --- |
| Group |  |  |  |  |
| No KMC | 1.00 | 1.00 | 1.00 | 1.00 |
| KMC | 1.61 (1.13, 2.31)* | 1.31 (0.92, 1.85) | 2.51 (1.74, 3.62)* | 2.61 (1.74, 3.93)* |
| Age |  |  |  |  |
| < 30 | 1.00 | 1.00 | 1.00 | 1.00 |
| 30-34 | 0.92 (0.62, 1.36) | 0.99 (0.67, 1.45) | 0.77 (0.51, 1.15) | 0.82 (0.51, 1.33) |
| >=35 | 0.99 (0.64, 1.53) | 0.93 (0.60, 1.44) | 0.59 (0.37, 0.93)* | 0.96 (0.55, 1.67) |
| Education attainment |  |  |  |  |
| High school | 1.00 | 1.00 | 1.00 | 1.00 |
| College | 0.97 (0.61, 1.55) | 1.15 (0.73, 1.82) | 0.77 (0.48, 1.24) | 0.74 (0.42, 1.29) |
| University & above | 1.01 (0.62, 1.63) | 1.67 (1.04, 2.69)* | 0.80 (0.49, 1.31) | 1.14 (0.64, 2.02) |
| Parity |  |  |  |  |
| Primipara | 1.00 | 1.00 | 1.00 | 1.00 |
| Multipara | 1.48 (1.05, 2.10)* | 1.08 (0.76, 1.53) | 1.55 (1.08, 2.23)* | 1.13 (0.73, 1.74) |
| Pregnancy-related complications |  |  |  |  |
| No | 1.00 | 1.00 | 1.00 | 1.00 |
| Yes | 1.37 (0.98, 1.93) | 0.76 (0.54, 1.06) | 0.86 (0.60, 1.21) | 0.55 (0.35, 0.86)* |
| Delivery mode |  |  |  |  |
| Vaginal delivery | 1.00 | 1.00 | 1.00 | 1.00 |
| C-section | 0.80 (0.57, 1.11) | 1.09 (0.78, 1.53) | 0.54 (0.38, 0.77)* | 0.93 (0.61, 1.42) |
| Birth weight |  |  |  |  |
| Normal (>=2500) | 1.00 | 1.00 | 1.00 | 1.00 |
| Low weight (<2500) | 0.76 (0.47, 1.24) | 0.56 (0.35, 0.90)* | 0.44 (0.26, 0.74)* | 1.21 (0.64, 2.27) |
| Gestational week |  |  |  |  |
| 36 weeks | 1.00 | 1.00 | 1.00 | 1.00 |
| Less than 36 | 1.53 (0.83, 2.83) | 1.32 (0.69, 2.51) | 1.75 (0.88, 3.48) | 2.08 (0.71, 6.13) |

Note: Additionally adjusted for occupation.
